# From Molecular Descriptors to Intrinsic Fish Toxicity of Chemicals: An Alternative Approach to Chemical Prioritization

**DOI:** 10.1021/acs.est.2c07353

**Published:** 2022-12-08

**Authors:** Saer Samanipour, Jake W. O’Brien, Malcolm J. Reid, Kevin V. Thomas, Antonia Praetorius

**Affiliations:** † Van ’t Hoff Institute for Molecular Sciences (HIMS), University of Amsterdam (UvA), 1090 GDAmsterdam, The Netherlands; ‡ UvA Data Science Center, University of Amsterdam, 1090 GDAmsterdam, The Netherlands; § Queensland Alliance for Environmental Health Sciences (QAEHS), The University of Queensland, Brisbane, QLD4072, Australia; ∥ Norwegian Institute for Water Research (NIVA), NO-0579Oslo, Norway; ⊥ Institute for Biodiversity and Ecosystem Dynamics (IBED), University of Amsterdam, 1090 GDAmsterdam, The Netherlands

**Keywords:** machine learning, LC_50_, QSAR, toxicity categorization, hazard assessment

## Abstract

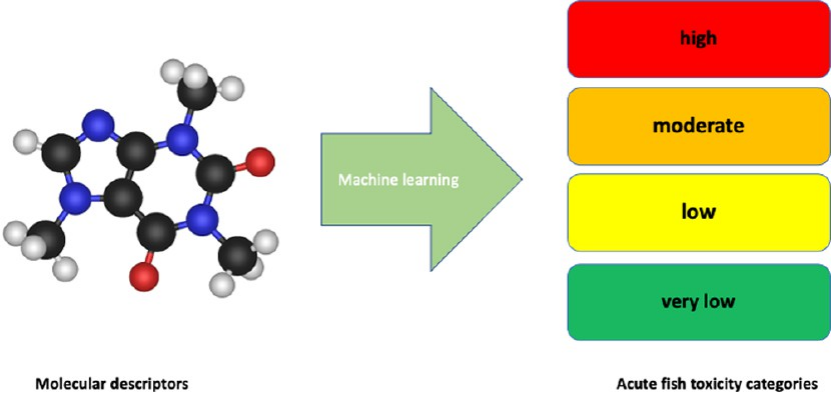

The European and U.S. chemical agencies have listed approximately 800k chemicals about which knowledge of potential risks to human health and the environment is lacking. Filling these data gaps experimentally is impossible, so *in silico* approaches and prediction are essential. Many existing models are however limited by assumptions (e.g., linearity and continuity) and small training sets. In this study, we present a supervised direct classification model that connects molecular descriptors to toxicity. Categories can be driven by either data (using *k*-means clustering) or defined by regulation. This was tested via 907 experimentally defined 96 h LC_50_ values for acute fish toxicity. Our classification model explained ≈90% of the variance in our data for the training set and ≈80% for the test set. This strategy gave a 5-fold decrease in the frequency of incorrect categorization compared to a quantitative structure–activity relationship (QSAR) regression model. Our model was subsequently employed to predict the toxicity categories of ≈32k chemicals. A comparison between the model-based applicability domain (AD) and the training set AD was performed, suggesting that the training set-based AD is a more adequate way to avoid extrapolation when using such models. The better performance of our direct classification model compared to that of QSAR methods makes this approach a viable tool for assessing the hazards and risks of chemicals.

## Abstract 2

In this study, a machine learning-based strategy that is an alternative to conventional quantitative structure−activity relationship models is used for the toxicity categorization of chemicals using molecular descriptors and direct classification.

## Introduction

The chemical space of the human exposome is ever expanding with a wider diversity of chemicals from the points of view of both fate and toxicity.^1−7^ The latest estimates of the numbers of environmentally relevant chemicals based on the chemical registries and production volumes are estimated to be between 350k and 800k.^2,8^ For most of these chemicals, there is little to no knowledge about their environmental fate or toxicity.^1−5,8,9^ Because the experimental assessment of the fate and toxicity of such a large number of chemicals is not feasible, modeling approaches to predict hazard indicators play an increasingly important role in chemical prioritization and risk assessment.^10−13^

Prediction of the physicochemical properties and biological activity (e.g., aquatic toxicity) has been one of the main approaches to dealing with the structural diversity in the chemical space.^10−13^ Most existing modeling strategies employ quantitative structure–activity relationship (QSAR) models and rely on building linear and/or nonlinear relationships between the structural descriptors and the modeled activity/property.^10,14−17^ These models are often built on very homogeneous training sets (i.e., similar chemical classes), hence the assumption of linearity.^17,18^ In fact, efforts have recently been spent on using more heterogeneous training sets as well as moving away from the assumption of linearity.^13,14,18,19^ Independent of the level of heterogeneity of the training data set, QSAR models are very limited in the number of measured activities as well as the number of chemicals evaluated (e.g., ∼1000 chemicals).^13,14,18,19^ The main consequence of this limitation is the fact that the models are used in extrapolation mode when used for prediction. This implies that the new data points are not represented adequately by the chemicals within the training set and are, thus, outside of the model applicability domain. The use of these models for extrapolation may result in very large prediction errors.^13,19,20^

For these predicted and measured activities (i.e., toxicity and/or other properties) to be translated into chemical management actions, they are divided into different categories using thresholds based on expert knowledge.^1,3,21−24^ Examples for such categories are environmental hazard categories defined by the Globally Harmonized System of Classification and Labeling of Chemicals (GHS) or thresholds for persistence (*P*), bioaccumulation potential (*B*), and toxicity (*T*) defined under the European Registration, Evaluation, Authorization and Restriction of Chemicals (REACH).^25^ The chemicals that fall within specific categories are then subjected to more active monitoring and eventually legislation.^24,26−28^ This process triggers wider experimental evaluation of chemicals within high-priority categories, which may result in the adjustment of the previously set thresholds, based on the new experimental evidence.^24,26,29^ However, for this chemical management strategy to be effective, a more accurate and reliable chemical prioritization (i.e., chemical categorization) approach is warranted.

In this study, we propose an alternative strategy for chemical prioritization on the example of acute aquatic toxicity, where the QSAR-based activity prediction step is skipped. Our direct classification model directly converts molecular descriptors into chemical categories, avoiding the errors inherent to the activity prediction step. As a proof of concept, this strategy was tested with experimentally determined 96 h lethal concentration (LC_50_) values for fish, for 907 organic chemicals. We compared the results of our direct classification strategy with the results of the conventional QSAR approach. Additionally, our modeling strategy was expanded to 32 000 chemicals from NORMAN SusDat.^27^ Finally, we performed a critical evaluation of applicability domains for all of the models in this study.

## Methods

### Overall Workflow

**Figure 1:**
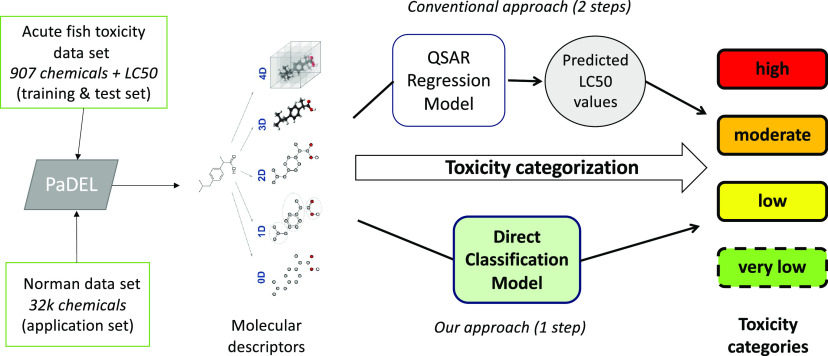
Overall workflow of the study from the raw data to the final generated models.

The data set used for our model development, validation, and testing consists of calculated descriptors, the monoisotopic mass of each chemical, and experimentally determined LC_50_ values (96 h) for acute fish toxicity (see details in Data Sets). The LC_50_ values were divided into four categories via *k*-means clustering: very low toxicity, low toxicity, moderate toxicity, and high toxicity. This categorization followed the typical evidence-based effect modeling categorization.^30−32^ Additionally, regulatory-defined toxicity categories were retrieved from the GHS. We assessed the prediction accuracy of the two types of toxicity categories by employing two different modeling strategies: a conventional QSAR regression model and direct classification (Figure 1). The QSAR regression model simulated the case in which the acute fish toxicity (as LC_50_) is predicted on the basis of molecular descriptors via a QSAR model and then the chemical is assigned a specific toxicity category in a separate step. On the contrary, the direct classification model skipped the LC_50_ prediction step and directly classified the chemical of interest into one of the initially defined toxicity categories. This comparison was performed for the full data set (i.e., training set and test set) to assess the accuracy of each approach in acute fish toxicity categorization.

### Data Sets

We employed two different data sets for our model development^18^ and model application.^33^ Our modeling data set consisted of experimental acute fish toxicity values for 907 chemicals retrieved from three databases, namely, OASIS, ECOTOX, and EAT5, and provided by Cassotti et al.^18^ The data consisted of the concentrations causing death in 50% of test fathead minnows (*Pimephales promelas*) over a test duration of 96 h (LC_50_ 96 h). More details regarding the data curation are provided elsewhere.^18^ We will refer to this data set as the “acute fish toxicity data set” hereafter. The chemicals in this data set covered different chemical families, including pharmaceuticals, pesticides, conventional persistent organic pollutants (POPs), and industrial chemicals. Throughout this article, we refer to the 907 chemicals with measured toxicity and curated descriptors as the full “acute fish toxicity data set”, the portion used for model development and validation as the training set, and the portion of the data used for additional model testing of the final model as the test set.

**Figure 2:**
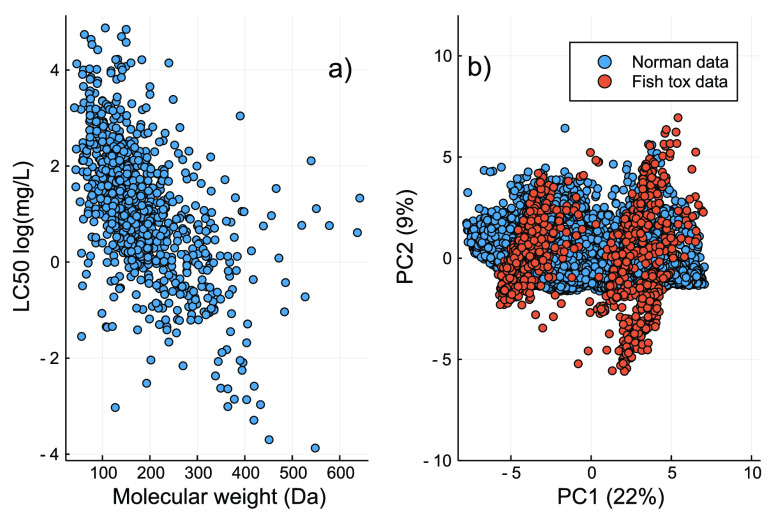
(a) Distribution of the experimental LC_50_ values used for model development and validation. (b) Chemical space via PCA covered by the acute fish toxicity data (i.e., training and test sets) and the NORMAN data set, where the curated descriptors were used for cluster analysis.

The second data set (hereafter termed the “NORMAN data set”) was an extract of ∼32 000 chemicals (31 722 chemicals), including their predicted 96 h LC_50_ values for acute fish toxicity (*P. promelas*) from the NORMAN SusDat database.^34^ This data set included only the chemicals that were reported to be within the applicability domain of the QSAR model developed by Aalizadeh et al.,^34^ which was used to test our model applicability (Figure 2). This is the model employed by the NORMAN Network for their risk assessment and chemical management. When checking the overlap between the acute fish toxicity data set and the NORMAN data set, we observed ∼100 common entries.

We calculated 2757 one-dimensional (1D) (i.e., constitutional/count descriptors), two-dimensional (2D) (i.e., structural fragments), and three-dimensional (3D) (i.e., graph invariants) molecular descriptors and PubChem fingerprints for both data sets using the PaDEL software package,^35^ implemented via a python 3 wrapper called padelpy. Additionally, the name of the chemicals, their SMILES,^36^ and their InChiKeys^37^ were retrieved from the PubChem database^38^ via pubchempy API. To identify the unstable descriptors caused by the lack of convergence during the structural optimization, we performed the descriptor calculations for the acute fish toxicity data set in triplicate. The descriptors were scaled by the maximum of each descriptor in the training set to minimize the impact of the descriptor magnitude on the final models.^39^ After scaling, the variance of each descriptor in the acute fish toxicity data set was calculated and only the descriptors that had a variance of <0.1 were kept. We assumed that the stable descriptors for the acute fish toxicity data set are also stable for the NORMAN data set. Therefore, the descriptors for this data set were calculated only once. Additionally, the maximum of each descriptor in the NORMAN data set was compared to those from the training set (from the acute fish toxicity data set). The descriptors with ratios of >100 were considered unstable and removed from both data sets, resulting in a total of 2036 final descriptors out of an initial 2780.

We also evaluated the coverage of the chemical spaces of the data sets by principal component analysis (PCA) (Figure 2). PCA is an unsupervised dimension reduction approach, which enabled us to assess the underlying trends in our data sets by combining several variables into a single principal component.^40^ To perform PCA, we used the curated descriptors matrix and in total two principal components.

### Toxicity Categories

To categorize the chemicals on the basis of their acute fish toxicity, we employed two different strategies: (1) applying *k*-means clustering to derive four categories from our acute fish toxicity data set and (2) using predefined categories for acute aquatic hazard as defined in the GHS.^41^

#### *k*-Means Clustering for Toxicity Categorization

The *k*-means strategy divided the chemicals into four categories consisting of high toxicity, moderate toxicity, low toxicity, and very low toxicity accounting for 96 h LC_50_ values for fish toxicity and monoisotopic masses of the chemicals. The *k*-means clustering algorithm is an iterative clustering algorithm, in which the distances between different measurements from a set of user-defined centers (so-called centroids) are used to cluster the data.^40^ This algorithm has the advantage of incorporating more than one parameter, compared to expert manual judgment in the clustering. Additionally, this algorithm, given that it has randomly selected centroids in the first iteration, requires further validation. Here we employed bootstrapping to ensure the selected acute fish toxicity categories (i.e., clusters) are robust enough for predictive purposes. To do that, the fish toxicity data were randomly divided into a 90% training set and a 10% test set. The training set then was bootstrapped with replacement for 500 iterations, to guarantee that each model is built on the basis of a unique data set. The most commonly identified centroid over 500 iterations was selected as the final model and for acute fish toxicity categorization. In the end, the final model was further tested using the test set. During the categorization, we provided the *k*-means algorithm with two variables, namely, 96 h LC_50_ values and monoisotopic masses, and four clusters, following the category structures adapted by previous studies.^30^

#### GHS Categorization for Acute Aquatic Hazards

In addition to *k*-means clustering, we also used the three categories for acute aquatic hazards of the GHS, which were hard set thresholds.^41^ The three GHS-based categories for short-term (acute) aquatic hazard are based on thresholds derived from 96 h LC_50_ values for acute fish toxicity: high toxicity (category acute 1, LC_50_ ≤ 1 mg/L), moderate toxicity (category acute 2, 1 mg/L < LC_50_ ≤ 10 mg/L), and low toxicity (category acute 3, LC_50_ > 10 mg/L) (see Table 4.1.1 in ref (42)).

### Modeling

In this study, we developed two different models: a QSAR regression model and a direct classification model. The details of each model strategy are provided below. Both models, once optimized with the acute fish toxicity data set, were used with the NORMAN data set to further assess their applicability.

#### QSAR Regression Model

We developed, optimized, validated, and tested a random forest regression model using the curated descriptors (independent variables) and the experimentally defined LC_50_ values (dependent variable). Random forest is a decision tree-based algorithm in which several bootstrap data (i.e., training set) are given to several decision trees. This assures that the data set given to each tree is unique.^40^ Once the model is developed, the most common decision tree model outcome is considered as the random forest model prediction. The main advantage of the random forest modeling strategy is the ability to handle nonlinearity and noncontinuity in the data, which is highly relevant to toxicity prediction.^43^ Here, the acute fish toxicity data set was divided into a training set (90% of the full data set) and a test set (10%). The training set was used for model development and optimization, while the test set was utilized for further evaluation of the data set. For the regression model, the model hyperparameter optimization was performed with a 2D grid with the number of trees ranging from 100 to 1000, whereas the minimum number of points in each leaf varied from 1 to 21. The combination of 3-fold cross-validation and the out-of-bag strategy enabled us to generate an optimized regression model while defining the importance of each variable. The variables that had relative levels of importance of >1% were considered as essential variables for the model. This strategy enabled us to quickly identify the variables most relevant to our model’s accuracy.

The final optimized regression model consisted of 600 trees, a minimum of four points in each leaf, and eight variables. This regression model was employed to predict the 96 h LC_50_ values for fish toxicity of the chemicals in the NORMAN data set. In a second step, the predicted LC_50_ values were used to categorize the chemicals into the two types of toxicity categories described above.

#### Descriptor-Based Direct Classification Model

We developed, validated, and tested a classification model to convert the curated descriptors into the acute fish toxicity categories. For this model, we employed random forest classification, implemented via the ScikitLearn.jl julia package.^44^

For the direct classification, we split the acute fish toxicity data set (i.e., curated the descriptors and toxicity categories) into a training set (90% of the full data set) and a test set (10%). To optimize the main model hyperparameters, the number of trees, and the minimum number of points in each leaf, we generated a grid with 20 steps for each parameter ranging from 200 to 2000 and from 1 to 21 for the number of trees and minimum data points in each leaf, respectively. For each model, we performed 3-fold cross-validation to systematically assess the model accuracy. The model with the highest cross-validation accuracy (i.e., 73%) was considered as the optimized classification model. This optimized classification model consisted of 1200 tress and a minimum number of points in each leaf of four. To avoid overfitting during the training process, when building the model, we set an out-of-bag cross-validation,^45^ in which only a randomly selected fraction (i.e., square root of the number of variables) of the variables was fed to individual trees. The combination of out-of-bag cross-validation and leaf purity was utilized to calculate the importance of individual variables to the final model. To select the relevant variables, we divided the variance explained by each variable by the largest one and selected those that contributed >1% to the model, thus 230 of 2036 variables.

To build the final model, the full acute fish toxicity data set was used with the selected variables. In this case, all of the selected variables were used for the final model building. Additionally, this model was used to categorize the NORMAN data set into the two types of acute fish toxicity categories directly based on the curated descriptors.

#### Applicability Domain

To assess whether a chemical is represented well by the model training set, we performed the applicability domain assessment. The applicability domain assessment was done by calculating the leverage of each chemical compared to the training set.^34^ The leverage was calculated using eq 1
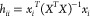
1where *X* is the matrix of the training set (including the descriptors), *x*_*i*_ is the vector of the descriptors for an individual chemical, and the *h*_*ii*_ is the calculated leverage. The leverage calculations are typically done only using the model variables, in other words only the descriptors used for the optimized model. In this study, we assessed both the full descriptor space (i.e., assuming the model using all of the descriptors) and the model specific descriptors (i.e., conventional approach). This strategy enabled us to systematically assess which chemicals are well represented by the training set.

### Calculations

All calculations were performed using a personal computer (PC) with an Intel Core i7 central processing unit and 16 GB of RAM operating Ubuntu 20.04.2 LTS. All of the data processing and statistical analysis were performed using julia language version 1.6.

## Results and Discussion

In this study, we developed a random forest-based direct classification model to convert the molecular descriptors of chemicals to predefined acute fish toxicity categories. This model was developed, validated, and tested via an experimentally defined data set of 96 h LC_50_ values for acute fish toxicity for 907 organic chemicals. The result of this strategy was directly compared to that of the conventional two-step approach, first QSAR-based property prediction and then toxicity categorization, for both the acute fish toxicity data and a data set of ≈32 000 chemicals from NORMAN SusDat.^33^

### Toxicity Categorization

**Figure 3:**
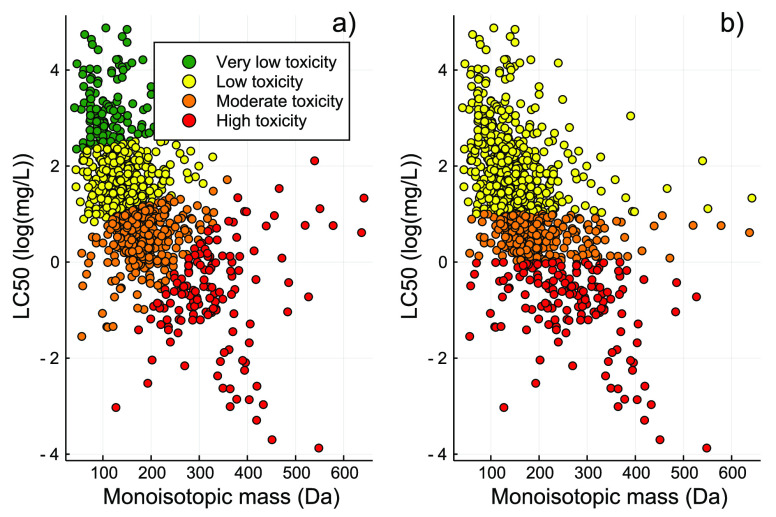
Distribution of the toxicity categories of the acute fish toxicity data set via (a) the best *k*-means clustering model and (b) based on GHS categories.

The final *k*-means model resulted in a clustering accuracy of 97.5%. This model was then fed the full acute fish toxicity data set to define the toxicity category of each chemical in that data set. The final model was saved as a binary file to be used for prediction (Figure 3). The *k*-means and GHS categories were used as labels in two separate runs of the direct classification model, while the 96 h LC_50_ values for acute fish toxicity predicted by the QSAR regression model were converted into the two types of acute toxicity categories in a second step.

When comparing the unsupervised *k*-means clustering-based categorization with the expert knowledge-based categorization from the GHS, we see a high level of similarity in the thresholds (Figure 3). In fact, the main differences were observed for chemicals with molecular weights of ≥400 Da and LC_50_ values of ≥1 mg/L [0 log(mg/L)]. These chemicals in the *k*-means categorization were considered part of the high-toxicity category, while on the basis of the GHS categories, they were considered moderate to low toxicity. When calculating the similarity scores between the descriptors of those chemicals and the two categories, we consistently observed higher values for the high-toxicity category. This indicates that those chemicals may be structurally more similar to the high-toxicity category rather than the moderate- and/or low-toxicity one. These similarities are better captured by the *k*-means model, given that it uses two variables (96 h LC_50_ and monoisotopic mass) and Euclidean distances for cluster creation.

### Performance of the QSAR Regression Model

**Figure 4:**
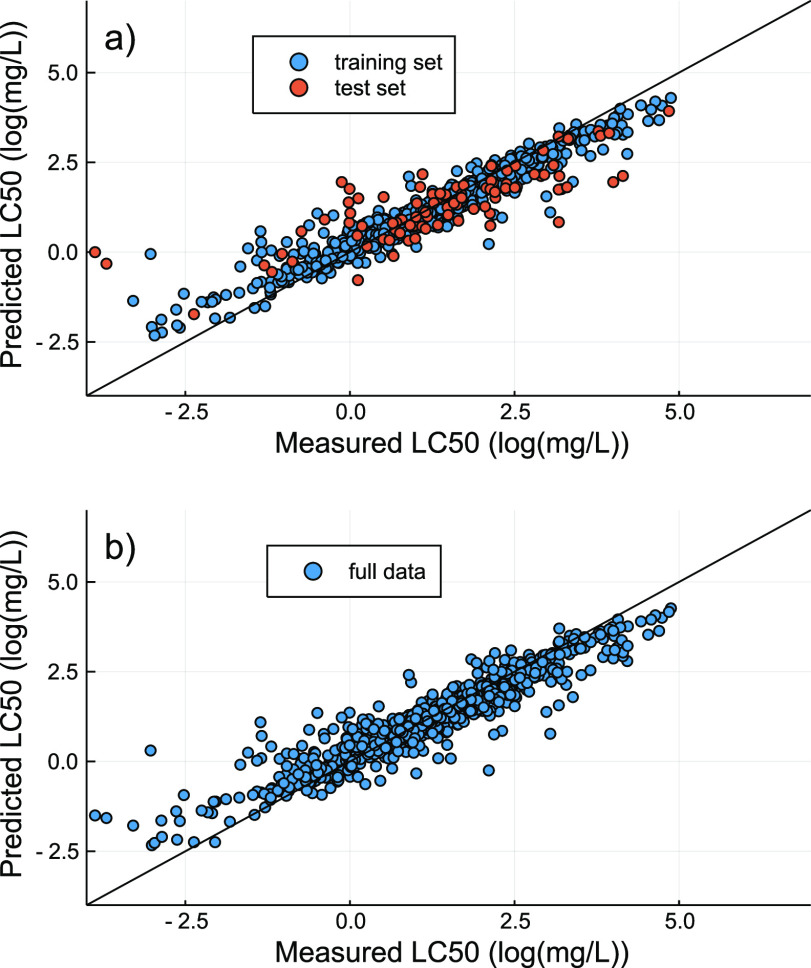
Measured vs predicted 96 h LC_50_ values for acute fish toxicity for (a) the training and test set during model optimization and (b) the optimized model with the full acute fish toxicity data set.

The residuals of the final and optimized QSAR regression model were between −1 and 1 in LC_50_ units for ≈95% of the data (Figure S2). This model consisted of 600 trees and eight variables, resulting in *R*^2^ values of 0.86 for the training set and ≈0.7 for both median cross-validation and the test set. The observed levels of accuracy were comparable to those of previously reported linear and nonlinear QSAR models^17,34^ (Figure 4). We observed up to 2.1 log(mg/L) overestimation of the LC_50_ for values of ≤−1, while our model resulted in a slight underestimation of toxicity for LC_50_ values of ≥5 (Figure 4 and Figure S2). Finally, we used the optimized model to predict the 96 h acute fish toxicity LC_50_ values for the NORMAN data set. When comparing the results of our predictions to the predictions by Aalizadeh et al.,^34^ we observed a clear linear trend (i.e., Pearson correlation coefficient of 0.68) between the two predictions, further indicating the validity of our model (Figure S3).

The optimized regression model included eight variables from which two were related to the logP of the chemicals in the training set (Figure S1). The most relevant variable was the Crippen logP^46^ value explaining ∼35% variance of the final model. This logP was calculated on the basis of 68 atomic contributions. On the contrary, the second variable was XlogP,^47^ implemented within PubChem.^38,48^ This logP calculation also uses the atomic contribution of 87 groups and additionally incorporates two correction factors, improving its accuracy and expanding its applicability. Another relevant variable for our regression model was the ZMIC1 descriptor, which is a 2D descriptor indicating the level of symmetry in a structure.^35^ Finally, the remaining relevant descriptors (i.e., excluding logP, XlogP, and ZMIC1 descriptors) were related to molecular connectivity, polarizability, and hydrogen-bond donation, which all have been shown to be relevant in explaining the physicochemical properties and toxicity of chemicals.^15,17,34^

### Performance of the Descriptor-Based Direct Classification Model

The optimized direct classification model resulted in a classification accuracy of 92% for the training set and ∼80% for both the cross-validation and the test set, for the four *k*-means categories. The final model used 230 variables out of a total of 2036 curated descriptors. Similar to the regression model, most of the important variables were a combination of 2D descriptors and fingerprints (i.e., 3D) (Figure S4). These descriptors included the four logP calculations (e.g., CrippenlogP) as well as parameters related to polarizability and charge distribution. These parameters are all highly relevant to the mobility of the chemicals and their binding potential with the active sites.^15,18^ As opposed to the regression model, the most relevant variable explained only ≈1.5% of the variance (vs 35% for the regression model) in the final model. Even though larger numbers of variables were included in the model, the total number of variables was <30% of the number of measurements, resulting in a mathematically well-defined problem. Additionally, a larger number of variables enables a better assessment of the model applicability domain.

The direct classification model based on the three GHS categories resulted in an accuracy of 94% for the training set and ∼85% for the cross-validation and test set. This model, similar to the previous one, had 236 highly important variables that were included in the final model. The highly important variables (e.g., top 20) for both models were exactly the same as for the direct classification into the *k*-means categories with similar levels of variance explained.

The reported statistics and the selected variables in our classification models further indicated the applicability of our model for the prediction of acute fish toxicity categories directly from the molecular descriptors.

### Classification versus Regression

The fish toxicity data were used to predict the toxicity categories via both the conventional QSAR regression model and the direct classification strategies. The QSAR regression model resulted in predicted LC_50_ values that were converted into the two types of acute fish toxicity categories in a subsequent step. In contrast, the classification model directly predicted the toxicity categories. The predicted acute fish toxicity categories based on both methods were compared to the true categories coming from the measured 96 h LC_50_ values for fish toxicity to evaluate the accuracy of each approach.

**Figure 5:**
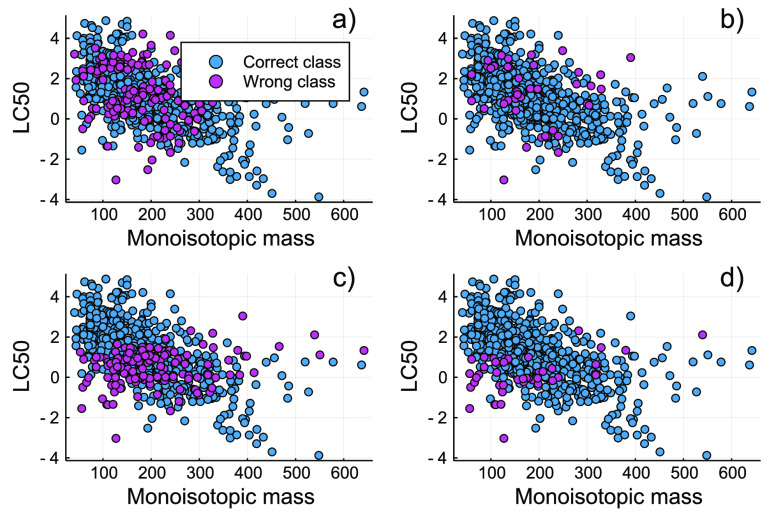
Correctly vs wrongly predicted acute fish toxicity categories based on (a) the QSAR regression model and *k*-means-based categories, (b) the direct classification strategy based on *k*-means categories, (c) the QSAR regression model using the GHS categories, and (d) the direct classification strategy with GHS categories.

The direct classification method, for both cases, resulted in ∼4 times fewer misclassifications when compared to the QSAR regression model. We observed 47 cases of misclassification for the *k*-means-based categories and 41 cases for GHS categories. This was in agreement with our expectations, given that the total numbers of classes in GHS categories were smaller, thus affording a lower probability of wrong classification. For the QSAR regression model, we observed 178 cases of wrong classifications for *k*-means-based categories whereas 163 incorrectly classified cases were observed for the GHS categories (Figure 5). The direct classification strategy showed a homogeneous distribution of the miscategorized chemicals in the acute fish toxicity data set, for the *k*-means and GHS categories. For the *k*-means categorization, the QSAR regression model resulted in a large and homogeneous distribution of wrong categorization, while for the GHS approach, we observed a high density of miscategorization for high- and moderate-toxicity groups (Figure 5).

Approximately 85% of the chemicals miscategorized via direct classification overlapped with those wrongly categorized via the QSAR regression model, regardless of the type of categories. For example, a chemical that was consistently wrongly categorized by all of the methods was 1-hydroxypyridine-2-thione (InChyKey, YBBJKCMMCRQZMA-UHFFFAOYSA-N) with a measured LC_50_ of 0.95 μg/L [i.e., −3.02 log(mg/L)]. This chemical was categorized as moderately toxic by both models but is actually a high-toxicity chemical. Upon examination of the structure of this chemical, it is clear that this chemical is not very well covered by our training set. In other words, there are not enough (at least four) chemicals with a structure similar to this one in our training set. This further indicates that the addition of more diverse chemical structures to our training set will result in even more accurate prediction of the toxicity categories. Additionally, the replacement of the molecular descriptors with the topographical fingerprints,^49^ given their stability, may further improve our prediction accuracy.

When comparing the distribution of the wrongly categorized chemicals, we observed higher levels of homogeneity in the *k*-means categories than in the GHS ones. This was consistent for both the QSAR regression model and the direct classification model. We also observed that for the GHS categories, the QSAR regression-based and direct classification models showed a high density of wrong categorization for chemicals at the border between the high- and moderate-toxicity regions. We interpret that this is mainly caused by the larger number of categories and lower levels of rigidity in the *k*-means approach compared to hard set thresholds (i.e., GHS approach).

The predicted LC_50_ values using our optimized QSAR regression model followed by the *k*-means clustering categorization resulted in 81% consistent classification between the acute fish toxicity categories generated by the direct classification method (Figure S5). However, the predicted LC_50_ values using the model developed by Aalizadeh et al.^34^ resulted in only 37% consistent toxicity categories. This may be due to the fact that our QSAR regression and direct classification models both had the same training set as well as the fact that our QSAR regression model uses eight descriptors while the model of Aalizadeh et al. uses only six (three of which are logP values).

Overall, our direct classification strategy showed a better performance in identifying the acute fish toxicity categories of the chemicals directly from the molecular descriptors, rather than passing via a QSAR regression model. We also observed a higher level of consistency between the categories generated by our models compared to that for another prediction method (i.e., Aalizadeh model). We interpret that the main reason behind the overall better performance of the direct classification approaches is first and foremost the fact the uncertainties associated with the QSAR regression models do not impact the categorization. Additionally, the inclusion of a larger number of descriptors in such models implies that higher levels of structural features are incorporated. In fact, the low level of variance explained by individual variables further confirms this hypothesis. Our direct classification model can be easily adapted to different types of predefined (acute fish toxicity) categories, as demonstrated here by classifying the chemicals following the categories for a short-term (acute) aquatic hazard of the GHS. Overall, these results indicate the viability of the classification strategy as a means of chemical prioritization and management.

### Applicability Domain

**Figure 6:**
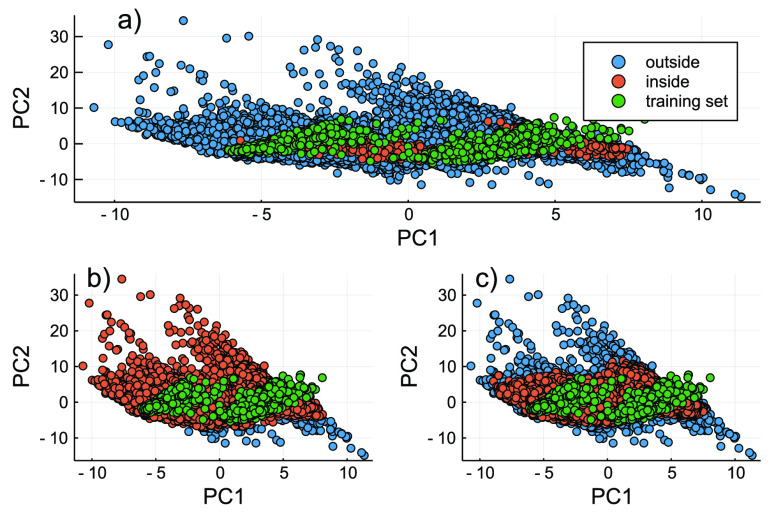
Applicability domain (AD) assessment (i.e., leverage calculation) of the NORMAN data set, based on (a) the training set (i.e., the full molecular descriptor space), (b) the QSAR regression model, and (c) the direct classification model. The blue circles represent the chemicals that are outside of the AD, the orange circles those within the model applicability domain, and the green circles those within the training set applicability domain.

We also evaluated the impact of AD selection for the assessment of the model coverage of the chemical space. To perform such an assessment, we calculated the leverage for the full descriptor space, QSAR regression model descriptors, and the direct classification model descriptors. Figure 6 depicts the plots of the scores for the training set and the NORMAN data set and the associated applicability domains.

With the full descriptor space (i.e., the curated descriptors used for our model development), only 585 entries of the NORMAN data set were covered by the training set. Using the regression model descriptors (i.e., the nine most relevant ones) resulted in ∼31 000 entries being covered by the training set. On the contrary, on the basis of the descriptors of the direct classification model, ∼27 000 entries were covered by the chemical space of the training set. The observed trend is in agreement with our expectations, given that the larger number of descriptors provides a better coverage of different structural characteristics of the chemicals. Upon examination of the chemical space covered by the training set (i.e., 96 h LC_50_ for acute fish toxicity) and the chemicals within the AD of the training set (i.e., the full descriptor space), a good level of overlap is observed. This is not the case when looking at the model specific ADs, implying an extrapolation with a much larger level of prediction error. An example of such cases is carbonothioylbis(iminomethylene) bis(diethyldithiocarbamate) (InChyKey, SPQBHESGHZSSMQ-UHFFFAOYSA-N), which was covered by the regression model AD and was not covered by the classification or training set AD. In fact, this chemical was one of the most different chemicals compared to the chemicals in the NORMAN data set (i.e., PC1 −11 and PC2 28). Therefore, it may be advisible to use the training set AD (i.e., the full descriptor space) to assess the training set coverage of the chemical space, rather than the individual model ADs.

## Implications for Chemical Assessment

The results of our direct classification model showed its power in categorizing the chemicals in terms of their acute fish toxicity based on their specific molecular descriptors. Our strategy can overcome the continuity assumption of QSAR models, which are conventionally used to fill experimental data gaps in the chemical assessment of structurally similar compounds, directly impacting the size of the training set. In other words, with our direct classification approach the experimental data sets from different sources and for different chemical families can be grouped to generate larger training sets resulting in more accurate predictions. As demonstrated here with the direct classification of the chemicals in the NORMAN data set into hazard categories defined by the GHS (based on acute fish toxicity), our approach can be adapted to different predefined categories as prescribed by various international regulations and/or classification or labeling systems. The direct classification approach can be expanded to other hazard categories (e.g., chronic toxicity) as well as to fate (e.g., mobility or persistence) and shows great potential for improving *in silico* tools for chemical hazard and risk assessment.

## Supporting Information Available

Details related to the types of samples, parameter settings for the models, and the figures (S1–S5) associated with the algorithms (PDF)

## Notes

The open access/source julia package for performing these calculations is available with MIT license at https://bitbucket.org/SSamanipour/toxcatpred-jl/src/main/. Additionally, all of the scripts for the model building are available in the same Bitbucket repository. Finally, the predictions of both models and all three ADs are available for download and use via FigShare (fish toxicity, 10.21942/uva.20089751; NORMAN SusDat, 10.21942/uva.20089787; and model output, 10.21942/uva.20089805). padelpy: https://github.com/ecrl/padelpy. pubchempy API: https://pubchempy.readthedocs.io/en/latest/. ScikitLearn.jl: https://scikitlearnjl.readthedocs.io/en/latest/.
