# Supplementary material for: From Molecular Descriptors to Intrinsic Fish Toxicity of Chemicals: An Alternative Approach to Chemical Prioritization
Source: Environ Sci Technol. 2022 Dec 8;57(46):17950–8. doi: 10.1021/acs.est.2c07353 (PMC10666547; doi:10.1021/acs.est.2c07353)
Supplement: Supplementary file 1 — es2c07353_si_001.pdf [file es2c07353_si_001.pdf]

Supporting Information for: From Molecular Descriptors to Intrinsic Fish Toxicity of Chemicals: an Alternative Approach to Chemical Prioritization

Saer Samanipour,*,†,‡,¶ Jake W. O'Brien,¶ Malcolm J. Reid,§ Kevin V. Thomas,¶ and Antonia Praetorius*,‖

†*Van 't Hoff Institute for Molecular Sciences (HIMS), University of Amsterdam (UvA), 1090 GD Amsterdam, the Netherlands*

‡*UvA Data Science Center, University of Amsterdam, 1090 GD Amsterdam, the Netherlands*

¶*Queensland Alliance for Environmental Health Sciences (QAEHS), The University of Queensland, Brisbane Qld 4072, Australia*

§*Norwegian Institute for Water Research (NIVA), NO-0579 Oslo, Norway*

‖*Institute for Biodiversity and Ecosystem Dynamics (IBED), University of Amsterdam, 1090 GD Amsterdam, the Netherlands*

E-mail: s.samanipour@uva.nl; a.praetorius@uva.nl

Number of pages: 6

Number of figures: 5

Number of tables: 0

S1 Regression Model

The figures S1 and S2 show the variables used for the final regression model building and the distribution of the residuals for that model.

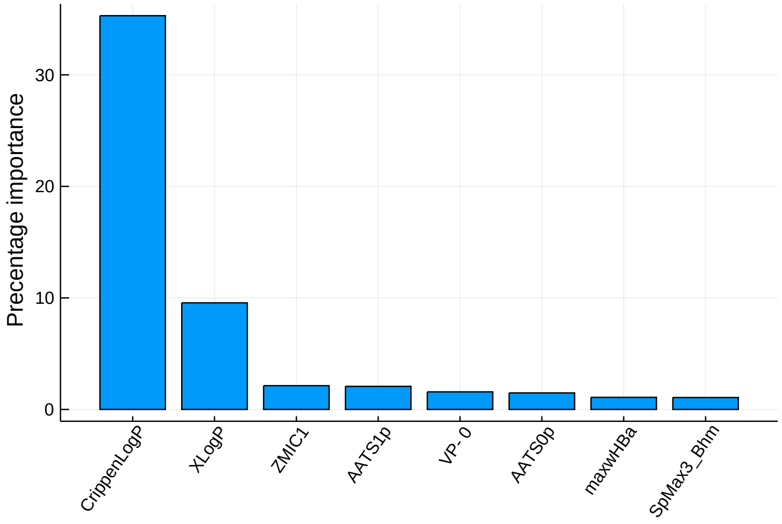

Figure S1: The most important variables included in the regression model.

S2 Classification Model

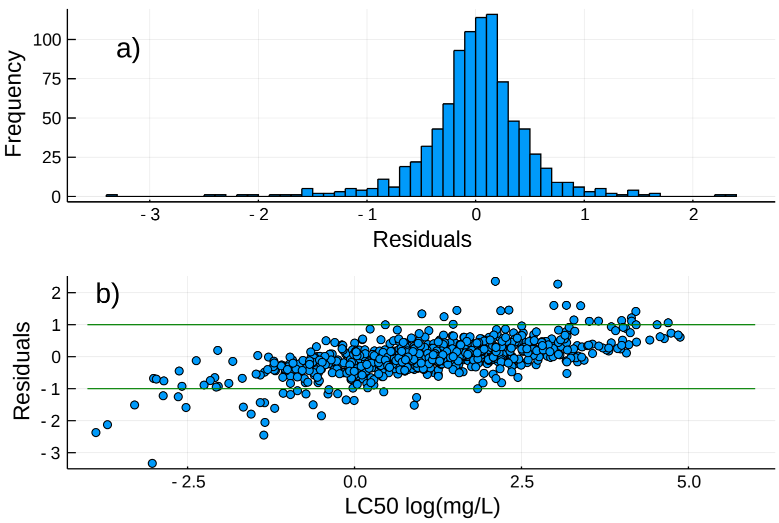

Figure S2: Shows a) the distribution of the the residuals of the regression model and b) depicts the residuals vs the LC50 value.

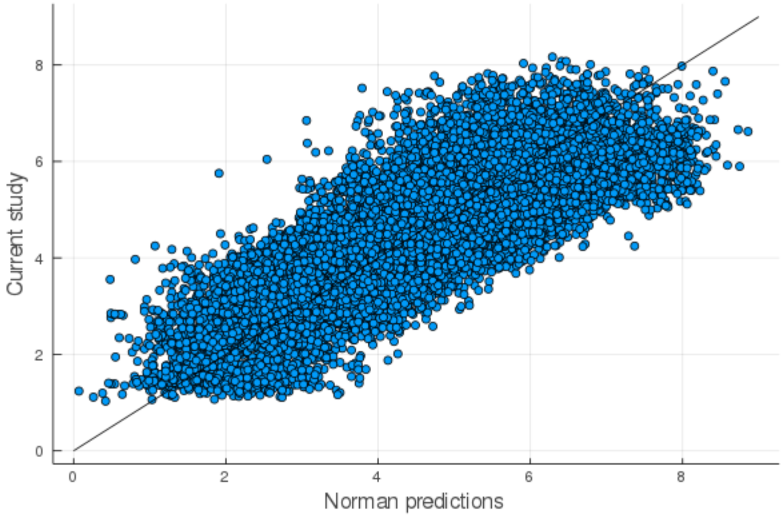

Figure S3: Shows the correlation between the predicted LC50 values by Norman model (Aalizadeh et al[1]) and our regression model for the SusDat data set.

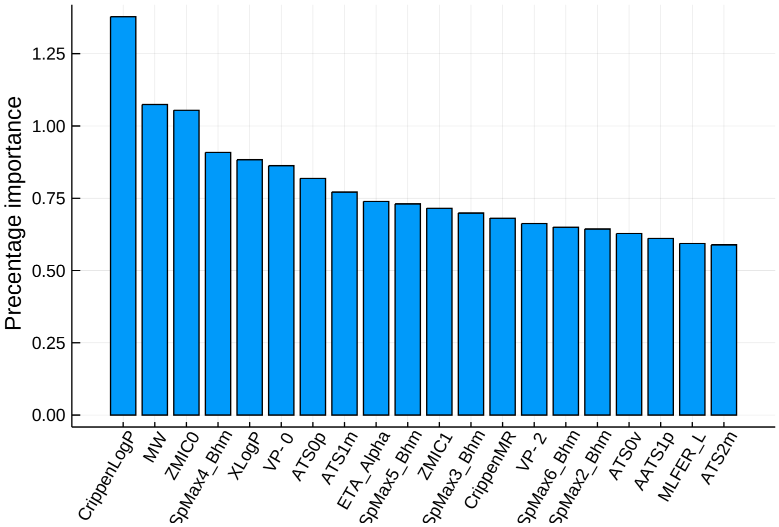

Figure S4: The top 20 most relevant variables used for the direct classification model building.

S3 Model Comparison

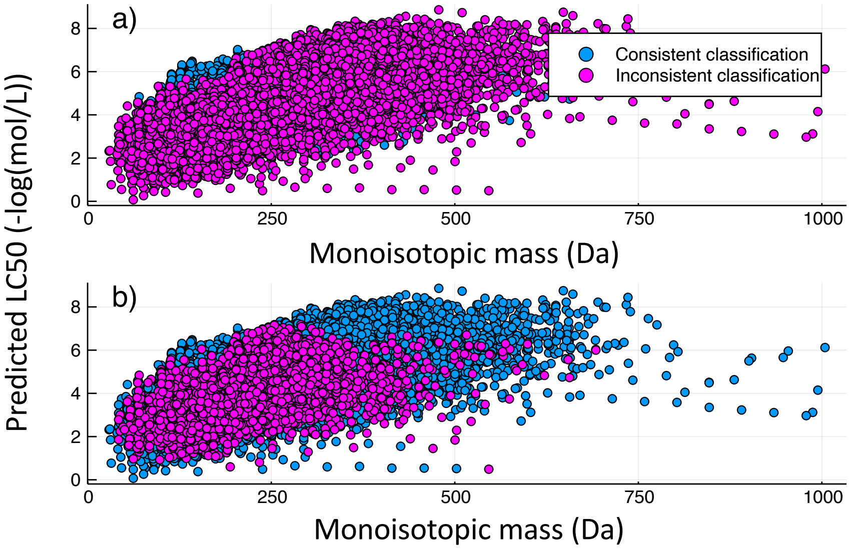

Figure S5: The direct comparison of the toxicity categories of the predicted LC50 (-log(mol/L)) a) via the model by Aalizadeh et al[1] and b) the current study, compared to the direct classification method.

References

(1) Aalizadeh, R.; Peter, C.; Thomaidis, N. S. Prediction of acute toxicity of emerging contaminants on the water flea Daphnia magna by Ant Colony Optimization–Support Vector Machine QSTR models. *Environmental Science: Processes & Impacts* **2017**, *19*, 438–448.
